# Epigenetic Regulation of Endothelial Cell Function by Nucleic Acid Methylation in Cardiac Homeostasis and Disease

**DOI:** 10.1007/s10557-020-07019-4

**Published:** 2020-08-04

**Authors:** Adam Russell-Hallinan, Chris J. Watson, Denis O’Dwyer, David J. Grieve, Karla M. O’Neill

**Affiliations:** Wellcome-Wolfson Institute for Experimental Medicine, School of Medicine, Dentistry and Biomedical Sciences, Queen’s University Belfast, Belfast, UK

**Keywords:** Epigenetics, Endothelial cells, DNA methylation, RNA methylation, Heart failure

## Abstract

Pathological remodelling of the myocardium, including inflammation, fibrosis and hypertrophy, in response to acute or chronic injury is central in the development and progression of heart failure (HF). While both resident and infiltrating cardiac cells are implicated in these pathophysiological processes, recent evidence has suggested that endothelial cells (ECs) may be the principal cell type responsible for orchestrating pathological changes in the failing heart. Epigenetic modification of nucleic acids, including DNA, and more recently RNA, by methylation is essential for physiological development due to their critical regulation of cellular gene expression. As accumulating evidence has highlighted altered patterns of DNA and RNA methylation in HF at both the global and individual gene levels, much effort has been directed towards defining the precise role of such cell-specific epigenetic changes in the context of HF. Considering the increasingly apparent crucial role that ECs play in cardiac homeostasis and disease, this article will specifically focus on nucleic acid methylation (both DNA and RNA) in the failing heart, emphasising the key influence of these epigenetic mechanisms in governing EC function. This review summarises current understanding of DNA and RNA methylation alterations in HF, along with their specific role in regulating EC function in response to stress (e.g. hyperglycaemia, hypoxia). Improved appreciation of this important research area will aid in further implicating dysfunctional ECs in HF pathogenesis, whilst informing development of EC-targeted strategies and advancing potential translation of epigenetic-based therapies for specific targeting of pathological cardiac remodelling in HF.

## Introduction

Heart failure (HF) is a complex clinical syndrome arising from any structural or functional abnormality, resulting in inadequate cardiac output to support physiological demand. HF is the leading cause of hospitalisation in patients aged > 65 years, carrying considerable economic cost and regarded as a major public health burden [1]. The HF syndrome is classified according to ejection fraction, natriuretic peptide levels and presence of structural heart disease and diastolic dysfunction into three subtypes: HF with reduced ejection fraction (HFrEF), HF with preserved ejection fraction (HFpEF) and HF with mid-range ejection fraction (HFmrEF) [2]. In the developed world, HF prevalence is ~ 1–2% of the adult population, but rises to > 10% after 70 years of age [2], and alarmingly, is predicted to increase by ~ 50% by 2030 due to an ageing population and increased prevalence of comorbidities including obesity, diabetes and hypertension. While therapeutic advances are encouraging, in particular for acute cardiovascular events, the mortality rate for HF patients remains high with 14% mortality within six months of diagnosis [3, 4].

HF is initiated by acute or chronic injury to the myocardium and evokes a diverse and complex array of cellular responses involving resident cardiomyocytes, fibroblasts, and endothelial cells (ECs), and infiltrating immune cells, which initially repair damaged tissue and maintain cardiac function. Over time, exaggerated wound-healing responses including cardiomyocyte hypertrophy, fibrosis and inflammation lead to aberrant remodelling and cardiac dysfunction [5–8]. Such remodelling, which manifests clinically as changes in size, shape and function of the heart, represents a key determinant of HF progression. A critical player in this HF pathogenesis is EC dysfunction, especially in the context of HFpEF. In addition, it is well established that pathological changes in HF are associated with altered regulation of gene expression. Over the last decade, extensive efforts have been made to define the role of epigenetic regulation, including DNA methylation, histone modifications and non-coding RNAs, in cardiac development and disease. While beneficial effects of epigenetic-modifying therapies on aberrant cardiac remodelling have been reported in several pre-clinical studies [9–12], the precise cellular targets of these agents are currently unknown. Whilst the role of DNA methylation and histone modification in cardiomyocytes, cardiac fibroblasts and immune cells has been comprehensively reviewed [13], emerging evidence suggests that modification of RNA bases, termed epi-transcriptomics, are important regulators of cellular behaviour with dysfunctional changes specifically implicated in HF pathogenesis [14]. Considering the increasingly apparent crucial role that ECs play in cardiac homeostasis and disease and predominant emphasis on epigenetic regulation in other cardiac cell types, this review will specifically focus on the role of nucleic acid methylation (both DNA and RNA) in the failing heart emphasising the key influence of these epigenetic mechanisms on EC function. For a full list of abbreviations, please see Table 1.

**Table 1:** Abbreviations

5aza	5-Azacytidine	hm6A	N6-Hydroxymethyladenosine
5azadC	5-Aza-2-deoxycytidine	HMEC	Human microvascular endothelial cell
5hMeC	5-Hydroxymethylcytosine	HNRNP	Heterogeneous nuclear ribonucleoprotein
5MeC	5-Methylcytosine	HREC	Diabetic human retinal endothelial cell
ADK	Adenosine kinase	HSPC	Haematopoietic stem and progenitor cells
ALKBH5	Alkylation repair homologue 5	HUVEC	Human umbilical vein endothelial cell
AngII	Angiotensin II	ICAM	Intercellular adhesion molecule
C38	Cytosine 38	ICM	Ischaemic cardiomyopathy
CGI	CpG islands	IGF2BP1–3	Insulin-like growth factor 2 mRNA-binding proteins 1–3
CpG	Cytosine-(phosphate)-guanine dinucleotide	lncRNA	Long non-coding RNA
CRISPR	Clustered regularly interspaced short palindromic repeats	m5C	5-Methylcytosine (RNA)
DCM	Dilated cardiomyopathy	m6A	N6-Methyladenosine
DNA	Deoxyribonucleic acid	MBD	Methyl-CpG-binding domain protein
DNMT	DNA methyltransferase	MCEC	Murine cerebral endothelial cell
DR	Diabetic retinopathy	MeCP	Methyl-CpG-binding protein
EC	Endothelial cell	METTL3	Methyltransferase like 3
ECFC	Endothelial colony-forming cell	MI	Myocardial infarction
ECM	Extracellular matrix	MMP	Matrix metalloproteinase
EDHF	Endothelium-dependent hyperpolarising factor	mRNA	Messenger RNA
EHT	Endothelial-haematopoietic transition	NO	Nitric oxide
eIF	Eukaryotic initiation factor	NSUN	NOL1/NOP2/sun domain
EndoMT	Endothelial to mesenchymal transition	PLAC	Placenta-associated
eNOS	Endothelial nitric oxide synthase	POLG	Polymerase γ-1
EPCs	Endothelial progenitor cells	Pprc2a	Proline-rich coiled-coil 2A
ET-1	Endothelin 1	R7W-MP	Cell-penetrating mimetic peptides
EZH2	Enhancer of zeste homologue 2	RNA	Ribonucleic acid
f6A	N6-Formyladenosine	RNAi	RNA interference
FTO	Fat mass and obesity–associated protein	ROS	Reactive oxygen species
HCAEC	Human coronary artery macrovascular endothelial cells	rRNA	Ribosomal ribonucleic acid
HCMEC	Human coronary microvascular endothelial cells	SAM	S-Adenosylmethionine
HF	Heart failure	siRNA	Small interfering RNA
HFmrEF	HF mid-range ejection fraction	TAC	Transaortic constriction
HFpEF	HF with preserved ejection fraction	TET	Ten-eleven translocation
HFrEF	HF with reduced ejection fraction	TGFB2	Transforming growth factor B2
HIF	Hypoxia inducible factor	TNFα	Tumour necrosis factor alpha
hm5C	5-Hydroxymethylcytosine (RNA)	tRNA	Transfer RNA
hm6A	N6-Hydroxymethyladenosine	Tsp-1	Thrombospondin-1
HMEC	Human microvascular endothelial cell	UTR	Untranslated terminal region
HNRNP	Heterogeneous nuclear ribonucleoprotein	VDAC1	Voltage-dependent anion-selective channel 1
HREC	Diabetic human retinal endothelial cell	VE	Vascular endothelial
HFpEF	HF with preserved ejection fraction	VEGF	Vascular endothelial growth factor
HFrEF	HF with reduced ejection fraction	vWF	Von Willebrand factor
HIF	Hypoxia inducible factor	WTAP1	Wilms’ tumour-associated protein 1
hm5C	5-Hydroxymethylcytosine (RNA)	YTH	YT521-B homology

## Endothelial Cells in Health and Disease

ECs line the interior of blood and lymphatic vessels, forming a metabolically active barrier between the circulation and smooth muscle, and playing a key role in the regulation of physiological processes, such as angiogenesis, wound healing, smooth muscle cell proliferation, fibrosis and inflammation [15]. The endothelium displays diverse structural and functional heterogeneity which adapts to the requirements of the host organ [16]. Growth of the vasculature occurs early in development with EC differentiation forming a primitive vascular network which is influenced by local environment and neighbouring cells, resulting in generation of ECs with tissue-specific functionality [17]. Regulation of vascular tone by ECs ensures appropriate delivery of blood to specific organs which is responsive to various stimuli, including shear stress, temperature and medications, and is largely mediated by nitric oxide (NO) and endothelium-dependent hyperpolarising factor (EDHF) [18, 19]. Disruption in normal physiology, due to factors such as hyperlipidaemia, physical inactivity, obesity, age, hypertension and poor diet [20], may result in EC dysfunction, reducing the ability of these cells to maintain cardiovascular homeostasis [21]. Specifically, oxidative stress (e.g. due to increased generation of reactive oxygen species [ROS] or reduced levels of endogenous antioxidants) and inflammation may lead to abnormal NO metabolism and bioavailability, resulting in characteristic imbalance observed in dysfunctional ECs [22]. This pathogenic microenvironment initiates a series of damaging events (such as cytokine activation and leukocyte adhesion), which promote vascular permeability and infiltration of inflammatory mediators and oxidised lipoproteins, leading to impaired integrity of the vascular wall, smooth muscle cell proliferation, inflammation and atherosclerosis [20].

It is well established that EC dysfunction plays a central role in HF development and progression [23], whilst EC paracrine signalling (e.g. NO, prostacyclin, angiotensin II [AngII] and endothelin 1 [ET-1]) is a critical modulator of cardiomyocyte survival, growth and contractility, and cardiac fibroblast function and extracellular matrix (ECM) turnover. Indeed, recent focus on ECs as the leading cellular driver of cardiac dysfunction in HFpEF represents a paradigm shift towards understanding a complex disease which currently has no clinically effective treatment that can reduce mortality [24]. A particular feature of HFpEF appears to be cardiac microvascular dysfunction [25–27]. For example, reduced coronary microvascular density, or microvascular rarefaction, was reported in myocardial tissue from HFpEF patients which was associated with increased fibrotic burden [25]. While the precise underlying mechanisms contributing to microvascular rarefaction in HFpEF are not fully understood, several factors are implicated, including loss of EC mass, due to apoptosis or differentiation, EC angiogenic dysfunction and inflammation [26–28]. Indeed, the prospective multi-centre, PROMIS-HFpEF trial reported that microvascular dysfunction was associated with disease severity in the absence of significant macrovascular coronary artery disease [27]. Collectively, these studies highlight EC dysfunction and loss of vascular structure likely drivers of myocardial hypoxia and consequent aberrant remodelling in HF.

Mounting evidence indicates that epigenetic pathways play a vital role in regulating key EC genes, such as *NOS3* (eNOS) [29], and are responsive to a wide range of intrinsic and environmental stimuli, including those involved in the pathogenesis of HF. Whilst it is widely appreciated that modification of histone proteins and regulation through non-coding RNAs contribute to EC homeostasis and dysfunction, this review will focus on the emerging role of DNA and RNA methylation in HF and their specific influence on EC function in response to pathological stimuli such as hyperglycaemia and ischaemia.

## DNA Methylation in Heart Failure

DNA methylation is a key epigenetic modification which involves covalent attachment of a methyl group to the carbon 5′ position of a DNA cytosine ring resulting in the formation of 5-methylcytosine (5MeC). This process mainly occurs in palindromic cytosine-(phosphate)-guanine dinucleotides (CpGs) throughout the genome, with ~ 10% of all CpGs clustered together in regions known as CpG islands (CGI). CGIs are on average a thousand base pairs in length, have GC-rich content, and are associated with ~ 70% of annotated mammalian promoters [30–32]. DNA methylation is mediated by DNA methyltransferase (DNMT) enzymes (DNMT1, DNMT3A and DNMT3B) using S-adenosylmethionine (SAM) as the methyl donor. DNMT1 is involved in the maintenance of established DNA methylation through preferentially binding to hemi-methylated DNA, allowing daughter cells to retain their parent cells’ methylation profile during cell division [33], whilst DNMT3A and DNMT3B mediate de novo DNA methylation. These enzymes are highly expressed in early embryonic cells, when the majority of programmed de novo methylation events occur, and become downregulated after differentiation and in adult somatic tissues [34].

**Fig. 1:**
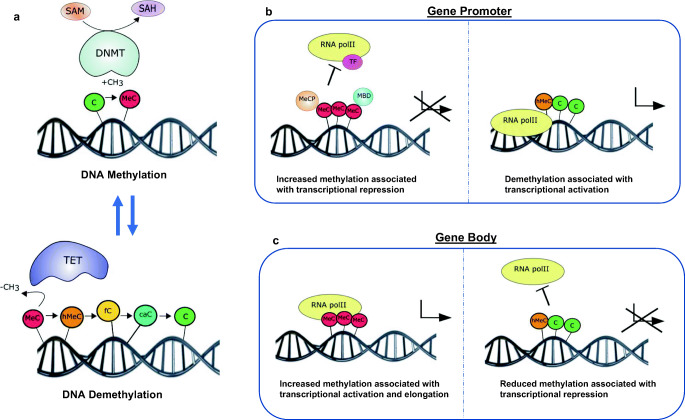
Gene regulation by DNA methylation and demethylation. **a** Cytosine residues (C) are methylated to 5-methylcytosine (MeC) under enzymatic action of DNMT (DNA methyltransferase) enzymes using SAM as a cofactor. Conversely, methylation marks can also be removed through demethylation; removal of methylated cytosines can be enzymatically carried out by the ten-eleven translocation (TET) enzymes, which oxidize MeC to 5-hydroxymethylcytosine (hMeC) and other breakdown products including 5-formylcytosine (fC) and 5-carboxylcytosine (caC). In the promoter region (**b**), increased DNA methylation prevents transcription factor (TF) and RNA polymerase II (RNApolII) binding to DNA and also recruits methyl-CpG-binding proteins (MeCP) and methyl-CpG-binding domain protein (MBD) proteins resulting in gene silencing. Removal of 5MeC from the promoter region therefore facilitates transcription. In the gene body (**c**), positive association has been demonstrated between increased methylation and transcriptional activation and elongation. Removal of 5MeC from the gene body therefore is associated with transcriptional repression

Regulation of gene expression by DNA methylation is primarily associated with transcriptional repression, occurring in the promoter region either directly or indirectly (Fig. 1). DNA methylation can directly interfere with the transcription factor binding to recognition sites in the promoter region [35]. Several transcription factors, such as the cyclic AMP-dependent activator CREB, HIF and NF-κB, recognise sequences containing CpG residues, and the binding of which to the promotor region is inhibited by methylation [35]. Indirect inhibition of gene expression can be due to recruitment of methyl-CpG-binding proteins, such as methyl-CpG-binding protein (MeCP) 1 and 2 and methyl-CpG-binding domain proteins (MBD1–4), to methylated DNA, promoting formation of a repressed chromatin state through interaction with co-repressor complexes (including components of other chromatin-modifying elements, histone deacetylases and histone methyltransferases). Interestingly, DNA methylation can also occur at intergenic regions and in gene bodies, with studies portraying a positive association between methylation and gene expression [36, 37] (Fig. 1). As with all epigenetic mechanisms, DNA methylation is dynamic in nature and can be enzymatically removed through oxidation of 5MeC to 5-hydroxymethylcytosine (5hMeC) by the ten-eleven translocation (TET) family of enzymes (TET1–3) (Fig. 1). Indeed, 5hMeC itself can act in a functional capacity by regulating gene expression rather than simply representing an intermediate breakdown product of 5MeC [38, 39].

A key role for DNA methylation in HF is becoming increasingly apparent, with numerous in-depth methylation profiling studies conducted to determine altered DNA methylation patterns in the heart and indicating key associations with cardiac disease and dysfunction across the different HF aetiologies [40–46]. Further to the first reported alterations in DNA methylation in patients with end-stage HF [40, 41], several major studies have specifically focussed on profiling DNA methylation changes in dilated cardiomyopathy (DCM) [42–44, 47]. Increases in CGI methylation in both *LY75* and *ADORA2A* were identified in DCM patients, whilst reduced expression of these genes promoted cardiac dysfunction and HF in zebrafish [42]. DNA hypermethylation was also found to be predominant in DCM, with > 90% of 158 differentially expressed genes associated with altered methylation patterns in their promoter regions, with further subtractive and computational analysis revealing four genes, *TK1*, *CLDN5*, *AURKB* and *BTNL9*, displaying both hypermethylation in their gene promoter and decreased expression in this setting [43]. Similarly, increased DNA methylation was reported in the left ventricle of DCM patients, highlighting several genes demonstrating an inverse relationship between methylation and expression (e.g. decreased methylation and increased expression of *HAND1* and *TBX5*, increased methylation and reduced expression of *FGF8* and *DNAJC10*) [47]. Interestingly, this particular study utilised right ventricular tissue from DCM patients as a proxy for normal cardiac tissue with meta-analysis of methylome data from the NIH Roadmap Epigenomics Project revealing similar methylation profiles between right ventricular samples from their DCM cohort and normal left ventricle [47]. Other groups have employed multi-omics approaches to identify epigenetic susceptibility regions and novel biomarkers linked to myocardial dysfunction in DCM. For example, one study identified 217 methylation sites that where altered between control and DCM patients which were detectable and conserved in both peripheral blood and cardiac tissue, specifically indicating hypomethylation of 5′-flanking CpGs of both *NPPA* and *NPPB* associated with elevated expression [44]. In the context of ischaemic cardiomyopathy (ICM), differential DNA methylation has been linked with differences in gene expression compared to non-ischaemic cardiomyopathy, indicating that ICM correlates with relative hypermethylation of promoter-associated CGIs associated with repression of genes involved in oxidative metabolism [45]. Indeed, across different HF aetiologies (including DCM, ICM and hypertrophic obstructive cardiomyopathy), 195 unique DNA methylation alterations have been identified in cardiac tissue in combination with associated gene changes [46]. When compared to normal cardiac tissue, subsequent gene expression analysis revealed 7 genes (4 hypermethylated [*HEY2*, *MSR1*, *MYOM3*, *COX17*], 3 hypomethylated [*PAIP1*, *CTGF*, *MMP2*] and 3 microRNAs [1 hypermethylated, *miR-24-1*; 2 hypomethylated, *miR-21*, *miR-155*]), with significantly altered levels consistent with the direction of methylation identified in the particular HF patient sub-group [46]. There is also increasing evidence suggesting that 5hMeC generated by the action of TETs may play a functional role in remodelling processes in the heart [48, 49]. Collectively, these studies demonstrate that altered DNA methylation profiles are evident in HF, highlighting the likely important role of this key epigenetic modification in disease pathogenesis. Indeed, this is underlined by reports of beneficial effects of both nucleosidal (5-azacytidine [5aza] and 5-aza-2′-deoxycytidine [5azadC]) and non-nucleosidal (RG108) inhibitors of DNA methylation across different preclinical models of HF, including myocardial infarction (MI) [50], pressure overload/hypertension [9, 10] and catecholamine stimulation [51], which appear to exert differential anti-hypertrophic and anti-fibrotic actions independently of cardiac stress. While the precise mechanisms underlying such cardioprotective effects of DNA methylation inhibitors remain to be determined, these agents show clear potential for therapeutic targeting of pathological cardiac remodelling in HF.

## Role of DNA Methylation in Endothelial Cells

While DNA methylation changes have been predominantly examined in both clinical and preclinical cardiac tissue containing a mixed population of cells, a major recent focus in the field is towards identifying relative alterations in individual cell types and their contribution to cardiac dysfunction in HF. The majority of such in vivo studies have primarily focussed on DNA methylation changes in cardiomyocytes [10, 52, 53], with one group suggesting that regulation of disease-associated genes (e.g. *CTGF*, *NPPA* and *NPPB*) in cardiomyocytes sorted from end-stage HF patients may rather occur primarily through alteration of histone signatures [52]. A small number of studies have explored DNA methylation changes in the other cardiac cell populations in response to injury. With regard to ECs, which represent the most abundant cell type in the heart, it is well known that several EC-specific genes, such as *NOS3* (encodes eNOS), *PECAM1*, CD31, vascular endothelial growth factor (VEGF) receptor 2 (*KDR*), von Willebrand factor (*vWF*) and vascular endothelial (VE)-cadherin (*CDH5*), display differential DNA methylation profiles in their promoter regions [54]. Several groups have studied the role of DNA methylation in the differentiation of ECs from embryonic stem cells, with particular interest in the potential application of epigenetic priming to improve efficiency [55, 56]. DNA methylation is also altered in ECs during ageing (genome instability/senescence) and upon exposure to high-glucose conditions (hyperglycaemia) or reduced oxygen supply (hypoxia/ischaemia). This review will specifically focus on DNA methylation alterations in ECs in the context of hyperglycaemia and ischaemia (Table 2), which are major drivers of cardiac remodelling and dysfunction associated with HF.

**Table 2:** Role of DNA and RNA methylation in endothelial cells

Stress	Cell type	Reported change(s)	Functional impact	Reference
DNA methylation				
Hyperglycaemia	HUVEC and Ea.hy926	Decreased 5MeC at *TGFB2* promoter	Increased activation of TGFB2 signalling	[57]
Hyperglycaemia	HREC	Increased DNMT1 levels and DNMT activity	Increased EC proliferation, VEGF and reduced antioxidant expression	[58]
Hyperglycaemia	Primary ECs isolated from muscle	Hyper- and hypo-methylation at genomic loci	Changes at genes regulating cell proliferation, growth and adhesion, cell-cell signalling	[59]
Hyperglycaemia	Fetoplacental AEC and VEC	Hyper- and hypo-methylation at genomic loci	Changes at genes regulating cell morphology and movement (actin organisation)	[60]
Hyperglycaemia	BREC	Increased 5MeC at *PLOG1* promoter	Decreased *PLOG1* (catalytic subunit of the mitochondrial DNA replication enzyme) expression, effecting ability to bind to mtDNA	[61]
Hyperglycaemia	HMEC/primary ECs from diabetic wound site (mouse)	Reduced DNMT1/DNMT3A with reduced methylation at *miR-200b* promoter	Increased *miR-200b* expression associated with impaired EC function	[62]
Hyperglycaemia	BREC	Increased TET2 binding increased 5hMeC at *MMP9* promoter	Increased 5hMeC and *MMP9* expression is associated with hyperglycaemia-induced mitochondrial damage	[63]
Hyperglycaemia	ECFCs	Reduced 5MeC at *PLAC8* promoter	Increased *PLAC8* expression associated with increased cellular senescence and reduced ECFC proliferation	[64]
Hyperglycaemia	ECFC	Reduced TET3 expression	ECFCs from diabetic patients are known to be dysfunctional	[65]
Hypoxia	HUVEC	Increased 5MeC at *NOS3* and MBD2 recruitment	Reduced *NOS3* expression associated with impaired cell survival and impaired angiogenic potential	[66]
Hypoxia	HCAEC/HCMEC	Increased 5MeC at *RASAL1* promoter (DNMT3A mediated)	Endothelial-to-mesenchymal transition and loss of EC phenotype	[67]
Hypoxia	HUVEC	Decreased DNMT activity decreases in global 5MeC at promoters of key angiogenic genes	Changes associated with increased angiogenic capacity	[68]
Anoxia and glucose deprivation	MCEC	Increased 5MeC at *THBS1*	Decreased *THBS1* expression (anti-angiogenic protein) demethylation and levels rescued with reoxygenation	[69]
Perturbed flow	HUVEC	Increased expression and nuclear translocation of DNMT1 associated with DNA hypermethylation.	Changes in DNA methylation associated with flow may be associated with promotion of atherosclerosis	[70]
Perturbed flow	HUVEC	Increased DNMT1	DNMT change associated with increased inflammatory potential of ECs	[71]
Perturbed flow	HUVEC	Increased DNMT1 and hypermethylation associated with non-reversed flow	Differential methylation occurring in genes associated with cellular metabolism, nucleic acid turnover and transcription associated with flow changes. Increased expression of DNMT1 with non-reversed flow reduces monocyte adhesion to ECs	[72]
Perturbed flow	HAEC	Increased DNMT3A with methylation at *KLF4* promoter	Reduced expression of *KLF4* which has effects on downstream EC function targets (e.g. eNOS)	[73]
RNA methylation				
Hypoxia	HUVEC	No changes in m6A levels and FTO expression	No reported impact on cellular function	[14]
Exposure to proinflammatory mediators	HUVEC	Increased efficiency of NSUN2 and methylation at the 5′ and 3′ UTR of *ICAM1*	Upregulation of *ICAM1* expression and increased inflammatory cell adhesion	[74]
Hyperglycaemia and oxidative stress	HUVEC	Upregulation of NSUN2 protein expression with increased cytosine methylation at the 5′ and 3′ UTR and coding region of *SHC* mRNA	Enhanced expression of *SHC* lead to increased ROS and premature cellular senescence	[75]

## Hyperglycaemia-Induced Endothelial Cell Dysfunction

Exposure of ECs to increased glucose levels leads to disruption of normal physiology, promoting initiation and retention of a pathophysiological state [76–78]. An important aspect of EC function is their ability to promote angiogenesis, which involves proliferation, migration, growth, differentiation and critical cell signalling events [79], and underlies tissue homeostasis and repair. In particular, diabetes-associated hyperglycaemia is known to cause diabetic cardiomyopathy, which appears to be driven by EC dysfunction [80]. Unlike histone modifications, whose role in diabetic ECs is well established [81, 82], the impact of DNA methylation in this setting is less well explored. However, several studies indicate a link between DNA methylation status in high-glucose exposed ECs and intrinsic function [57, 59, 60, 83]. For example, gene ontology analysis of transcriptomic data from ECs isolated from ischaemic muscles of diabetic or hyperlipidaemic mice highlighted a number of genes involved in pathways directing cell proliferation, growth and adhesion, cell-cell signalling and nutrient responses which displayed parallel changes in expression and promoter cytosine methylation [59]. Another report indicated that two umbilical vein EC lines, human umbilical vein ECs (HUVECs) and Ea.hy926 ECs exposed to high glucose levels displayed reduced DNA methylation at their promoter regions together with activation of transforming growth factor B2 (TGFB2) signalling, which is strongly implicated in the pathophysiology of diabetes and associated organ fibrosis [57]. Confirmation of an emerging key role for global gains in DNA methylation in mediating EC functional response to hyperglycaemia was provided by a report that hyperproliferation in diabetic human retinal ECs (HRECs) was attenuated by co-culture with the nucleosidal DNA methylation inhibitor, 5aza, in association with reduced pro-angiogenic VEGF expression and restoration of endogenous antioxidant levels [58]. Of further relevance to diabetic retinopathy (DR), hypermethylation at the promoter of polymerase γ-1 (*POLG*), involved in DNA replication, was demonstrated in both diabetic retinal tissue and cultured bovine retinal ECs [61]. Additional alterations in DNA methylation of multiple genes in parallel with atypical tissue morphology, actin organisation and barrier function have been reported in ECs isolated from diabetic placenta, with pathway analysis highlighting particular association with genes clustered to cell morphology and movement functions [60]. Consistent with the concept of intra- and inter-EC heterogeneity [16, 84–86], arterial and venous ECs were reported to display varying sensitivity to high glucose exposure, with the former showing DNA methylation changes in 408 genes whilst the latter exhibited alterations in only 159 genes [60]. DNA methylation may also play a role in hyperglycaemia-mediated regulation of small non-coding RNAs in ECs, as human microvascular endothelial cells (HMECs) were found to show reduced methylation at the *miR-200b* promoter in response to high glucose, in parallel with decreased expression of both DNMT1 and DNMT3A expression, effects which were reversed upon treatment with either a miR-200b inhibitor or the methyl donor, SAM, which also restored EC function. Of direct clinical relevance, these findings where confirmed in mouse ECs isolated from diabetic wounds, whilst administration of SAM to diabetic animals limited *miR200b* expression and improved wound perfusion [62].

Endothelial colony-forming cells (ECFCs) are a distinct subset of endothelial progenitors that circulate in the blood and contribute to blood vessel development and repair. Their specific role in neovascularisation continues to drive interest in their therapeutic (both autologous and allogenic) potential for vascular disease [84, 87, 88]. Although it is well established that these cells are largely dysfunctional in diabetic patients [89, 90], only a small number of studies have reported alterations in their DNA methylation status following hyperglycaemic exposure [64]. One study of ECFCs isolated from diabetic placental tissue found that increased levels of *PLAC8* (Placenta-Associated 8), linked with impaired proliferation and increased senescence, were correlated with reduced DNA methylation at 17 CpG sites, alterations which were reversed following RNAi depletion of *PLAC8* [64], highlighting a possible role for this protein and associated DNA methylation changes in hyperglycaemia-induced ECFC dysfunction.

Further studies have linked DNA methylation with glycaemic memory [91, 92], with the idea that persistent alterations in levels of this modification may continue to drive EC dysfunction after cells are removed from a hyperglycaemic environment. In support of this suggestion and despite diabetic patients following an established treatment regime (exercise or insulin), decreased DNA methylation at the *PLAC8* promotor (and increased expression) was found to persist in isolated ECFCs with impaired proliferative capacity and a senescent state [64]. Furthermore, hypermethylation of the *POLG1* promoter and increased DNMT activity in high glucose–treated HRECs persisted after reconstitution of normal glucose conditions for a 3-month period [61]. An additional and underexplored aspect of EC response to elevated glucose levels is the process of active DNA demethylation mediated by TET activity. Dysregulation of TET expression has been reported in ECFCs isolated from diabetic patients [65], whilst reduced DNA methylation, together with increased *TET2* expression and elevated levels of 5hMeC, at the metalloproteinase-9 (*MMP9*) promoter was observed in high glucose–treated HRECs. Notably, upregulation of MMP9 damages mitochondria and increases capillary cell apoptosis within the retina, thereby contributing to DR development, and siRNA knockdown of TET2 was found to attenuate increased 5hMeC and MMP9 levels, thereby protecting against high glucose–induced mitochondrial damage [63].

Taken together, these studies indicate that exposure of ECs to a hyperglycaemic environment leads to alterations in their DNA methylation status which contribute to attenuation of various aspects of EC function. This includes impacting their ability to orchestrate normal angiogenic growth and repair under pathophysiological high-glucose conditions. Expanding these DNA methylation studies into the specific context of the heart is required, where the detrimental effects of hyperglycaemia on myocardial ECs and vasculature are well recognised. Whilst further experimentation is also required to investigate how DNA methylation alterations in response to hyperglycaemia may be specifically manipulated, their potential as novel drug targets for both vascular disease (in the case of ECFCs) and HF is clear.

## Ischaemia-Induced Endothelial Cell Dysfunction

Lack of or restricted blood supply to myocardial tissue is central to promoting cardiac dysfunction in HF, with ischaemia and hypoxia specifically associated with enhanced ECM deposition, inflammation and cell death [93, 94]. As ECs are highly important in maintaining myocardial perfusion, they contribute to all clinical manifestations of HF. As with other cardiac cell types, ECs are susceptible to ischaemic injury which can impact their functional capacity. Hypoxia is known to impair EC integrity, resulting in increased vascular permeability and leukocyte infiltration [95]. More importantly, in the context of myocardial reperfusion, ECs can act as a double-edged sword by promoting blood flow whilst potentiating ischaemic cardiac damage through enhanced oxidative stress [96]. Indeed, it is appreciated that both hypoxia and ROS can promote alterations in DNA methylation in ECs, leading to changes in phenotype and function and disruption of vascular homeostasis.

Accumulating evidence suggests that ECs may undergo phenotypic change in response to hypoxia, known as endothelial-to-mesenchymal transition (EndoMT), in which cells adopt fibroblast-like properties. For example, both human coronary artery macrovascular (HCAEC) and HCMECs were reported to show a mesenchymal phenotype after 72 h in 1% oxygen (morphological changes, reduced CD31, elevated SNAIL, SLUG, TWIST, S100A4, and α-smooth muscle actin). This was associated with loss of EC phenotype and reduced expression of *RASAL1* due to increased DNMT3A-mediated CpG methylation in the promoter region [67], as previously shown in myocardium of end-stage HF patients [49]. Loss of vascular support in the heart due to EndoMT ultimately leads to enhanced myocardial hypoxia, thereby promoting further EndoMT, vascular loss and pathological cardiac remodelling. As such, retaining the EC phenotype through targeting DNA methylation may represent a novel approach to attenuate hypoxia-induced EndoMT in HF.

In response to hypoxia, activation of HIF-dependent EC signalling facilitates an adaptive angiogenic response to low environmental oxygen which appears to involve DNA methylation changes. For example, HUVECs cultured under hypoxia (0.5% oxygen) for 2 days demonstrated increased intracellular adenosine levels due to HIF-dependent reduction of adenosine-metabolising enzyme adenosine kinase (ADK). This was associated with increased angiogenic capacity and reduced DNMT activity and global hypomethylation at the promoter regions of key angiogenesis genes, including *KDR*, *NOS3*, *GATA4* and *SEMA5* [68]. In contrast, another study of HUVECs maintained in hypoxia for 16 h reported increased DNA methylation at the 5′-flanking region of *NOS3* that contains 13 CpG elements, leading to recruitment of MBD2 and repression of eNOS expression, which was reversed by targeted siRNA knockdown together with enhanced expression of *KDR* [66]. Complementary analyses in *Mbd2*-deficient mice demonstrated increased vascular expression of eNOS and KDR expression and improved recovery from experimental hindlimb ischaemia indicated by increased capillary and arteriole density versus wild-type controls [66]. This observation could at least partly explain why patients with vascular disease respond poorly to pro-angiogenic agents as hypoxia-induced DNA methylation changes, mediated by effector proteins such as MBD2, may promote EC dysfunction. Indeed, murine cerebral ECs (MCECs) exposed to both anoxia and glucose deprivation demonstrated increased DNA methylation at the promotor of thrombospondin-1 (*Thbs1*), a matricellular glycoprotein which promotes anti-angiogenic actions through inhibition of VEGF/NO signalling and EC migration/proliferation, which resulted in reduced Thbs1 expression at both RNA and protein levels [69, 97–99]. Interestingly, subsequent restoration of normal oxygen and glucose conditions for 4 h led to demethylation at the *Thbs1* promotor and restoration of gene expression, suggesting that these dynamic methylation changes are inherently reversible at the cellular level [69]. Conversely, culture of human dermal microvascular ECs (HMECs) in hypoxia (1% oxygen) for 24 h was shown to increase levels of *THBS1*, whilst treatment of normoxic ECs with hypoxic EC–conditioned media reduced EC proliferation and induced apoptosis [100]. Although this study did not investigate *THBS1* methylation changes, it is likely that differential culture conditions and exposure times (e.g. anoxic and glucose starvation for 4 h [69] versus 1% oxygen and normal glucose for 24 h [100]) and use of different ECs (primary MCECs versus immortalised HDMECs) may have accounted for contrasting results. While not conclusive, this work collectively suggests that DNA methylation may play an important role in regulating pleiotropic factors, such as THBS1, in unique populations of ECs under different environmental circumstances.

In addition to oxygen levels, vascular perfusion and direction of blood flow are known to regulate DNA methylation and gene expression in ECs, with several groups highlighting an important role for DNMT1 in modulating EC function in response to shear stress and differential flow. For example, increased nuclear DNMT1 expression together with enhanced levels of 5MeC was reported in ECs subjected to low and oscillatory shear stress using both in vitro and in vivo systems [70]. Similarly, elevated DNMT1 levels in response to perturbed blood flow were attenuated with 5aza treatment [71]. Other studies have also reported increased DNMT1-mediated DNA methylation in the context of both increased shear stress (induced in vivo by vascular occlusion or in vitro using cone and plate flow apparatus) and unidirectional flow, associated with global hypermethylation of genes associated with cellular metabolism, nucleic acid turnover and transcription [72]. Interestingly, and consistent with the previously described study, increased expression of DNMT1 observed under unidirectional flow resulted in reduced monocyte adhesion to ECs which was reversed by treatment with 5azadC in both in vitro and in vivo models [72]. Notably, when ECs maintained under shear stress were exposed to reversed flow, DNMT1 expression remained unaltered whilst monocyte adhesion to ECs was increased, prompting the authors to propose that hypermethylation under shear stress with unilateral flow may limit the arteriogenic capacity of ECs, thereby preventing hypertrophy/hyperplasia which may lead to impaired perfusion and tissue hypoxia [72]. In addition to DNMT1, other DNMT enzymes have been implicated in regulating EC function in response to altered blood flow. For example, increased DNMT3A levels are reported in HAECs subjected to disturbed flow for 2 days, linked with hypermethylation at the promotor of anti-inflammatory and anti-thrombotic, *KLF4*, and decreased binding and expression of myocyte enhancer factor-2 [73]. DNMT3A-mediated methylation negatively impacted on downstream functional targets of KLF4 such as *NOS3*, *THBD* and *MCP1*, which was rescued by treatment with pharmacological inhibitors of DNMT (RG108 and 5aza) [73].

Collectively, these studies highlight that both hypoxia and abnormal perfusion dynamics can influence DNA methylation in ECs resulting in changes to their normal physiology and behaviour, thereby promoting EC dysfunction. Whilst there is clear potential for specifically targeting this epigenetic process in ECs for treatment of HF, particularly in the context of ischaemic cardiomyopathy, further investigation is required to define underlying mechanisms.

## RNA Methylation

While methylation of cytosine bases on DNA is an established gene regulatory mechanism in the field of epigenetics, it is increasingly appreciated that modification of RNA bases is also critical to many aspects of gene expression including RNA export, assembly, translation and stability [101]. Since these modifications can fundamentally change the RNA properties without altering the transcript sequence, this emerging field of molecular biology investigating effects of RNA modifications on cell and organ function has been termed epi-transcriptomics. Compared with DNA, the range of modifications present in RNA is much higher, with > 160 different types reported [102], which are classified into two broad categories, substitutional or non-substitutional, depending on whether the modification impacts the nucleotide sequence. The role of substitutional modifications, such as A-to-I RNA editing, in cardiovascular disease has been extensively reviewed elsewhere [103] and is not the focus of this article. Of non-substitutional RNA modifications, methylation is the most studied and prevalent, with evidence suggesting a critical role in the regulation of cellular homeostasis. Five types of RNA methylation have been identified, namely N1-methyladenosine, N6-methyladenosine (m6A), 5-methylcytosine (m5C), N7-methylguanine and 2-O-methylation. Methylation of RNA bases involves a dynamic interplay between a variety of different writer, eraser and reader proteins. This research area is attracting increased attention, largely due to the development of high-throughput sequencing technologies, which has promoted understanding of the biological role of RNA methylation in cardiac homeostasis and disease. For the purposes of this review, the role of adenine and cytosine RNA methylation in HF will be specifically discussed, emphasising the key role which these modifications play in regulating EC function.

## Adenine Methylation in Endothelial Cells and Heart Failure

**Fig. 2:**
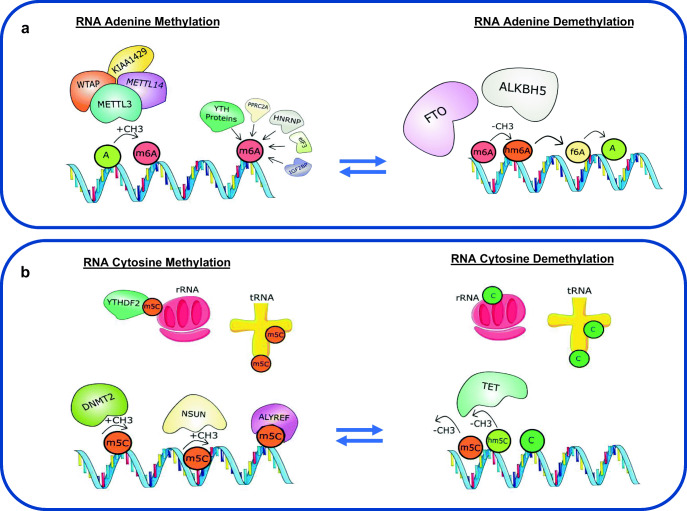
Dynamic regulation of adenine and cytosine RNA methylation and demethylation. **a** Adenine residues (A) are methylated (m6A) under enzymatic action of the methyltransferase complex comprising of methyltransferase like 3 (METTL3), METTL14, Wilms’ tumour–associated protein 1 (WTAP1) and KIAA1429. Proteins such as the YT521-B homology (YTH) family of proteins, heterogeneous nuclear ribonucleoprotein (HNRNP) protein, eukaryotic initiation factor 3 (eIF3), proline rich coiled-coil 2A (PPRC2A), insulin-like growth factor 2 mRNA-binding proteins (IGF2BPs) act as readers that recognize and bind to m6A. Conversely, m6A can also be removed through enzymatic demethylation by oxidation by both fat mass and obesity–associated protein (FTO) and alkylation repair homologue 5 (ALKBH5) to N6-hydroxymethyladenosine (hm6A) and N6-formyladenosine (f6A). m6A plays a critical role in regulating mRNA fate, including pre-mRNA splicing, mRNA stability, nuclear transport and translation. **b** Cytosine bases (C) in RNA can also become methylated by the methyltransferase action of the NOL1/NOP2/Sun (NSUN) domain-containing family and the DNA methyltransferase 2 enzyme (DNMT2), leading to formation of 5-methylcytosine (m5C) on different RNA species including messenger RNA, ribosomal RNA (rRNA) and transfer RNA (tRNA). Proteins such as ALYREF and YTHDF2 act as m5C reader proteins and recognize and bind to m5C on RNA. m5C marks can be removed via demethylation by the action of the ten-eleven translocation (TET) enzymes leading to the formation of hydroxy-m5C (hm5C). RNA cytosine methylation has been shown to influence numerous aspects of RNA biology, including structure, stability and translation of mRNA along with the biogenesis and function of ribosomes

Of all RNA modifications, the addition of a methyl group at position N6 of the adenine residue to form m6A is the most characterised and abundant post-transcriptional mRNA modification in eukaryotes. The formation and removal of m6A on both coding and non-coding RNA is a dynamic process which involves several writer and eraser proteins (Fig. 2a). The formation of m6A is mediated by a multicomponent methyltransferase complex comprising methyltransferase like 3 (METTL3) and METTL14 linked with Wilms’ tumour–associated protein 1 (WTAP1) and KIAA1429 [104]. METTL3 has been identified as the catalytic core of the complex [105] which requires other essential components to efficiently recognise, localise and methylate adenosine sites on RNA, thereby enabling m6A formation [104]. Further to recent developments in transcriptome-wide m6A mapping approaches (m6A immunoprecipitation coupled with high-throughput RNA sequencing), it is now appreciated that m6A modifications of RNA are conserved in both mice and humans, showing enrichment near stop codons and 3′ untranslated terminal regions (UTR) and within internal long exons [106, 107]. In mammals, roughly 0.1–0.4% of all adenosines in cellular RNA are m6A modified and account for more than 80% of all RNA base methylations [106–108].

Similar to DNA methylation, m6A can undergo enzymatic adenosine demethylation in RNA, with two enzymes identified as playing a key role, fat mass and obesity–associated protein (FTO) and alkylation repair homologue 5 (ALKBH5). These enzymes catalyse the removal of m6A through oxidation to N6-hydroxymethyladenosine (hm6A) and N6-formyladenosine (f6A) [109], with studies demonstrating a global increase in m6A in mRNA in response to targeted deficiency of RNA methylases [110, 111]. The intermediate products of m6A removal have a relatively short half-life under physiological conditions (~ 3 h), and it remains to be determined if hm6A and f6A serve specific biological functions [109].

Complementary to writer (establish m6A) and eraser (remove m6A) proteins, reader proteins, located in the cytoplasm and nucleus and identified through methylated RNA pulldown coupled with mass spectrometry, can recognise and read m6A methylation marks on mRNA and mediate the effects of m6A-modified transcripts [106]. Of these, the YT521-B homology (YTH) family of proteins is the most common and possesses an evolutionarily conserved YTH domain, YTHDF1–3, YTHDC1 and YTHDC2, that selectively recognises and binds to m6A [106, 112, 113]. Other proteins have also demonstrated the ability to recognise and bind directly to m6A sites, including members of the heterogeneous nuclear ribonucleoprotein (HNRNP) protein family [114], eukaryotic initiation factor (eIF) 3 [115], proline rich coiled-coil 2A (PPRC2A) [116], and insulin-like growth factor 2 mRNA-binding proteins 1–3 (IGF2BP1–3) [117]. The binding of these reader proteins to m6A sites is reported to regulate various aspects of mRNA fate, including pre-mRNA splicing, mRNA stability, nuclear transport and translation [118–123].

It is now appreciated that m6A methylation regulates structural and functional properties of RNA and that this modification is essential for maintenance of cellular homeostasis. Indeed, various studies have highlighted a critical role for m6A methylation in key biological processes such as embryonic and tissue development [124, 125], control of circadian clock [126], regulation of body weight and metabolism [127, 128], learning and memory [129], spermatogenesis [111, 113], response to stress [130, 131] and cellular renewal and differentiation [132–134]. It is therefore unsurprising that dysfunctional changes in this RNA modification are associated with pathological conditions, including various cancers [135], obesity and metabolic diseases [136], neurological disorders [137] and cardiovascular diseases, such as arrhythmia [138], coronary artery disease [136] and HF [14, 139, 140]. With regard to HF, increased m6A levels have been demonstrated in hearts of patients with DCM compared to those without [139]. Specifically, 1595 methylated transcripts were identified particular to DCM that were enriched in genes associated with cardiac development, transcription and cell adhesion, and reduced in genes involved in translation and protein targeting [139]. Similarly, global levels of m6A were found to be elevated in hearts of patients with ischaemic cardiomyopathy, which was also evident in multiple experimental ischaemia models (including mice and pigs) and linked with global reduction in cardiac FTO expression [14]. However, the association of other m6A writers (*Mettl3*, *Mettl4*, *Mettl14*) and erasers (*Alkbh5*) with ICM was inconsistent and with varying cardiac expression detected at different time points post-MI [14]. Interestingly, in the context of DCM, increased levels of m6A were not associated with altered expression of *FTO* or *METTL3* [139]. Whilst the precise molecular changes underpinning dysfunctional global m6A methylation are unclear, it is likely that variance reported in different HF aetiologies is influenced by underlying cardiac disease and risk factors mediating altered behaviour of the methylated transcripts. Indeed, this has been highlighted in the context of pressure overload, in which hearts from transaortic constricted (TAC) mice showed reduced m6A in mRNA relative to sham animals mirrored by decreased M*ettl3* gene expression 2 days after surgery [139].

To better understand the functional role of global m6A changes in the heart, much research has focussed on investigating the specific importance of this modification in individual cell types. As with DNA methylation, the majority of such studies have focused on defining alterations of the m6A machinery, through gain or loss of function, in cardiomyocytes, and its subsequent impact in cardiac homeostasis and disease. For example, *Fto* overexpression in cardiomyocytes exposed to hypoxia in vitro led to improvement in sarcomeric function and calcium signalling, whilst *Fto* knockdown caused aberrant cardiomyocyte function including altered repolarisation and increased arrhythmia [14]. Interestingly, siRNA knockdown of *Fto* in cardiomyocytes blunted cellular hypertrophy in response to α-adrenergic stimulation [139], suggesting that impact of m6A methylation on cellular function/behaviour may be dependent upon specific environment or stress. Indeed, AAV9-targetted overexpression of *Mettl3* in cardiomyocytes attenuated TAC-induced pathological cardiac remodelling (hypertrophy and fibrosis) after 2 weeks [139], whilst another group reported that transgenic overexpression of *Mettl3* in cardiomyocytes did not influence TAC-induced cardiac structural and functional changes after 8 weeks, but that *Mettl3* deletion in cardiomyocytes resulted in maladaptive eccentric cardiac remodelling in response to natural ageing and pressure overload [140]. Although further work is clearly required to elucidate the functional role of the cardiomyocyte m6A machinery, such studies support the concept that changes in m6A methylation of RNA may alter cellular behaviour and contribute to pathological changes.

As the majority of work to date has focused on cardiomyocytes, it is unclear whether changes in m6A levels or the enzymes that mediate this epi-transcriptomic process may regulate EC function in the healthy and/or diseased heart. Hypoxia has been previously highlighted as a key stimulus driving changes in m6A through HIF-dependent mechanisms in other cell types, including breast cancer stem cells in which ALKBH5-mediated demethylation of 3′ UTR m6A in pluripotent factors (including *NANOG*) is reported [141]. One study found that incubation of HUVECs in hypoxia for 16 h had no impact on *FTO* gene expression relative to cells exposed to normoxia, although expression of m6A components was not assessed [14]. Interestingly, however, the authors noted that mice with both transient and sustained AAV9-mediated cardiac-specific *Fto* expression demonstrated a higher number of CD31-positive ECs at the infarct border zone after 4 weeks post-MI. While the precise reason for this apparently enhanced angiogenic response at the border zone with *FTO* overexpression is unclear, it may be that cardiac manipulation of adenine demethylases induces pro-reparative or vasoprotective signalling in ECs, which could have therapeutic implications for HF where defects in the cardiac microvasculature (e.g. reduced capillary density) are an established hallmark of disease progression [25, 142, 143].

Mechanisms involved in dynamic acquisition and maintenance of cellular identity are critical for normal physiological development, whilst novel paradigms in developmental vascular biology indicate that during embryogenesis, haematopoietic stem and progenitor cells (HSPCs) are derived from a subset of ECs in the ventral wall of the dorsal aorta through a process known as endothelial-haematopoietic transition (EHT). This transitional process involves induction of a haematopoietic transcriptional programme in selected ECs resulting in tight-junction dissolution, loss of cell polarity and release into the circulation [144, 145]. As such, EHT is a complex, multi-step process that is critical for lifelong reconstitution of the blood. While the precise molecular mechanisms underlying EHT are not fully understood, it appears that m6A methylation may be critical in regulating this developmental transition. Simultaneous knockdown of *mettl3* and reader protein *ythdf2* in zebrafish embryos was found to prevent EHT, resulting in retention of EC phenotype characterised by increased expression of markers such as *tbx20*, *hey2* and *ephrinB2a* [146]. Furthermore, addition of m6A by mettl3 near the stop codon of the *notch1a* mRNA transcript in ECs facilitated binding of ythdf2, resulting in mRNA decay of *notch1a* and acquisition of HSPC phenotype (increased expression of *runx* and *cmyb*) [146]. This highlights a mechanism by which m6A methylation during embryonic development may repress Notch signalling, which is known to prevent EHT and enable retention of EC phenotype [132, 147]. Ultimately, these findings suggest that m6A methylation is an important regulator, maintaining EC fate during development, which could be potentially exploited for treatment of HF.

## RNA Cytosine Methylation

Similar to catalytic addition of methyl groups to cytosine residues on DNA, cytosine bases in RNA can also become methylated leading to the formation of 5-methylcytosine (m5C) (Fig. 2b). RNA methylation has been demonstrated on a variety of species including mRNA, rRNA, tRNA, snRNA, miRNA and lncRNA [148–150]. The ability to assess cytosine methylation in vitro and in vivo has been primarily facilitated through developments in methylation mapping approaches based on chemical derivatisation (bisulphite conversion or treatment with nucleosidal inhibitors e.g. 5aza) coupled with enrichment strategies (e.g. antibody-based immunoprecipitation) followed by RNA sequencing, as discussed in a recent review [148]. These approaches have been employed to identify m5C sites across different species. The abundance of m5C has been reported to range from 0.02 to 0.1% [151] with distribution found to be highly conserved in mammalian cells and tissues and enriched near the translation initiation sites of mRNAs [152–154].

Several different enzymes are responsible for the formation of m5C on RNA, including the seven-member NOL1/NOP2/Sun (NSUN) domain-containing family (NSUN1–7) and DNMT2, which utilise SAM as the methyl donor to form m5C. Whilst DNMT2 shares the same conserved catalytic motifs and a clear phylogenetic relationship as DNA methylating DNMTs [155, 156], it utilises this DNMT-like mechanism to act on RNA as a substrate to form m5C. Such RNA methyltransferase activity is demonstrated by its ability to methylate a small set of tRNAs at a specific site, cytosine 38 (C38), close to the anticodon [157], thereby increasing the tRNA stability and impacting protein translation [158, 159]. The remaining enzymes catalysing m5C belong to the NSUN family which mediate RNA methylation on the majority of RNA species through their active site bearing two catalytic cysteines [160]. Interestingly, it appears that specific levels of RNA cytosine methylation and activity of individual RNA methyltransferases may exert functional influence on cellular responses to drug treatment [161].

Formation of m5C has been shown to influence numerous aspects of RNA biology, including structure, stability and translation of mRNA [148], and biogenesis and functionality of ribosomes [162, 163]. Similar to adenine methylation, there are associated ‘m5C reader’ proteins which can mediate the activity of methylated RNA species, such as nuclear export factor ALYREF, which binds to and regulates nuclear export of mRNAs based on its m5C status [153]. Other m5C reader proteins have been described, including the m6A reader, YTHDF2, which can also recognise and bind to m5C within RNA to influence rRNA methylation. Additional putative m5C reader proteins include YTHDF1 and 3, pre-mRNA splicing factors, SFPQ and NONO, and mRNA cleavage stimulation factors, CSTF1–3, which have been identified in HeLa and HEK293T cells using SILAC-based quantitative proteomics [164]. Such work has highlighted a new concept of overlap between methylation reader proteins for different RNA methylation marks. While it appears that cross-talk between adenine (m6A) and cytosine (m5C) methylation marks influences both methylation and protein expression patterns [165], it is not known whether reader protein specificity for methylation marks is evident under physiological or pathological conditions.

As with removal of 5MeC from DNA, it is now appreciated that TET enzymes are also involved in catalysing hydroxylation of m5C to hydroxy-m5C (hm5C) on RNA, including mRNA [166]. While the precise functional role of hm5C on RNA is poorly understood, recent evidence suggests that 5hMeC may play an important role in post-transcriptional gene regulation in murine embryonic stem cells [167] and infection-induced myelopoiesis [168].

## RNA Cytosine Methylation in Endothelial Cells and Heart Failure

Similar to adenine methylation, alterations in enzymes that mediate cytosine methylation of RNA (in particular the NSUN family) are implicated in various pathological conditions including solid and haematological malignancies [169–171], and neurodevelopmental disorders such as autosomal recessive Dubowitz syndrome [172, 173]. Notably, RNA cytosine methylation occurs more frequently in mice, and to a lesser extent in human heart and muscle tissue, compared with other tissues (such as spleen), with m5C-enriched genes linked with mitochondrial function (e.g. voltage-dependent anion-selective channel 1, *VDAC1*) [149]. However, unlike m6A methylation, there are relatively few reports of RNA cytosine methylation in HF. Cardiac-specific deletion of *Nsun4* in mice resulted in cardiac hypertrophy after 5 weeks of age, characterised by impaired assembly of mitochondrial ribosomes and inhibition of mitochondrial translation [174]. Interestingly, pathological cardiac hypertrophy and fibrosis with preserved function was demonstrated in mice deficient in *Dnmt2*, associated with reduced cytosine methylation of the non-coding RNA, Rn7sk [175]. Absence of *Dnmt2* in embryonic stem cells also promoted RNA polymerase II activity with enhanced cardiac cell differentiation, suggesting that DNMT2 may be an important regulator of this process through methylation of non-coding RNAs such as *Rn7sk* [175].

The influence of RNA cytosine methylation on EC function is becoming increasingly apparent, especially in the context of metabolic dysfunction and inflammation. For example, NSUN2 is reported to methylate *ICAM-1* mRNA at 5′ and 3′ UTR in ECs exposed to pro-inflammatory mediators (tumour necrosis factor-α [TNF-α] and homocysteine), resulting in enhanced translation and protein expression both in vitro and in vivo and associated with increased leukocyte adhesion and formation of allograft arteriosclerosis, which was attenuated by genetic deficiency of NSUN2 [74]. Similarly, upregulation of NSUN2 in HUVECs exposed to hydrogen peroxide and high glucose led to increased cytosine methylation at the 5′ and 3′ UTR and coding region of *SHC* mRNA that was accompanied by premature senescence and enhanced expression of key proteins, such as *SHC*, *TP53* and *p16* [75]. There is also evidence to suggest that NSUN2 methylation may coordinate oxidative stress–induced senescence in epithelial carcinoma cells lines through synergistic effects with METTL3/METTL14-mediated m6A methylation [165], although it is yet to be determined whether cooperation of different RNA methylation marks contributes to EC dysfunction in the context of cardiovascular disease.

## Conclusion and Future Perspectives

**Fig. 3:**
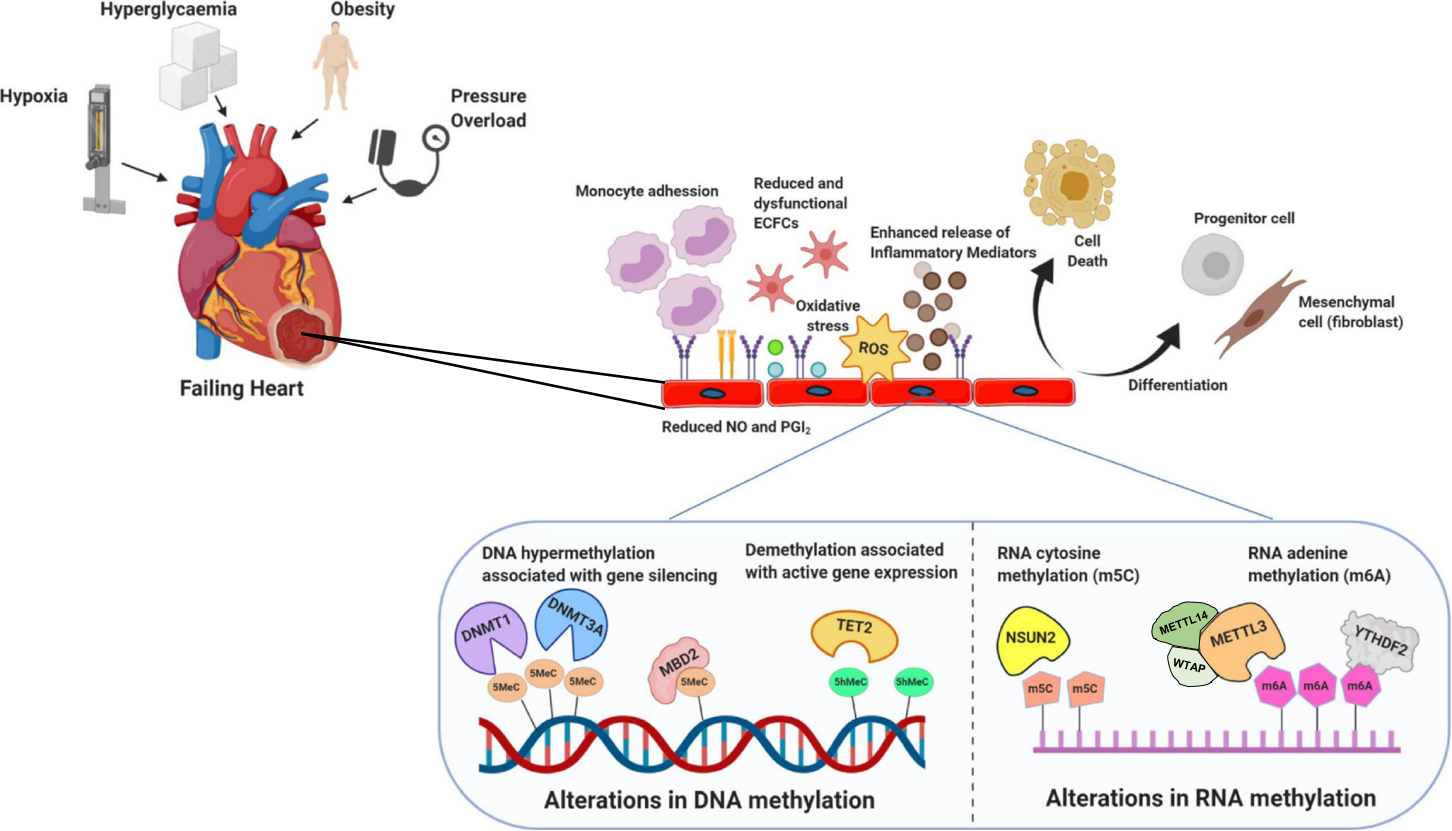
Schematic overview highlighting key changes in DNA and RNA methylation in endothelial cells that occur in response to cardiac stress promoting endothelial dysfunction and pathological remodelling in the heart. Endothelial progenitor cells (EPCs); nitric oxide (NO); prostaglandins (PGI2); reactive oxygen species (ROS); DNA 5-methylcytosine (5MeC); 5-hydroxymethylcytosine (5hMeC); DNA methyltransferase (DNMT); methyl-CpG-binding domain protein 2 (MBD2); ten-eleven-translocation 2 enzyme (TET2); NOP2/Sun domain family member 2 (NSUN2); RNA 5-methylcytosine (m5C); N6-methyladenosine (m6A); methyltransferase like 3 (METTL3); methyltransferase like (METTL14); Wilms’ tumour-associated protein (WTAP); YTH N6-methyladenosine RNA binding protein 1 (YTHDF2)

Remarkable progress has been made towards understanding molecular mechanisms underlying changes in both DNA and RNA methylation in cardiovascular development and disease, especially in the context of HF. Whilst the majority of these pioneering studies assessed DNA and RNA methylation in cardiac tissue, there is now a major shift towards dissecting the relative contribution of epigenetic changes within individual cell types in HF. In this regard, ECs line the entire vasculature where they perform an essential role in maintaining homeostasis; they are particularly important in the heart where they represent the largest cell population forming an active barrier between the coronary circulation and myocardium. It is therefore unsurprising that dynamic changes in nucleotide methylation in response to injurious stimuli, such as hypoxia and metabolic dysfunction (e.g. hyperglycaemia), have been implicated in promoting EC dysfunction and HF (Fig. 3).

Beyond the scope of this review, it is appreciated that DNA and RNA methylation interact with and form complex regulatory networks with other epigenetic modifications, including those to histone tails, ATP-dependent chromatin remodelling complexes, and other noncoding RNA species (miRNAs and lncRNAs), to dynamically modulate chromatin structure and gene expression. One example of such cooperation in the context of ICM is indicated by interaction between enhancer of zeste homologue 2 (EZH2, component of polycomb repressive complex 2 and a histone methyltransferase) and DNA methylation to regulate ischaemia-induced expression of metabolic enzymes and transcription factors in the heart [45]. With specific regard to cooperation of epigenetic processes in ECs, DNA methylation has been shown to regulate expression of miRNAs and lncRNAs in hyperglycaemia, thereby impacting EC function [62, 176]. Although not specifically determined in ECs, crosstalk of epigenetic processes has also been described for RNA methylation, whereby the histone modifications [177] and non-coding RNAs [178] are reported to act as guides for depositing m6A on RNA. The role of these epigenetic mechanisms, including histone modifications [133, 179], miRNAs [180, 181], lncRNAs [182], and RNA editing [103] in regulating EC (patho)physiology are comprehensively reviewed elsewhere.

It is well established that ECs within individual organs are not homogenous, with their expression profile and activity differing depending on arterial, venous, capillary, and sinusoidal origin. Such heterogeneity includes differences in cellular morphology, production of ECM, and tight junction formation [183–185], due to physical and biochemical alterations in the relative microenvironment, with arterial and venous ECs exposed to different levels of oxygen, waste products, blood flow and pressure [186]. It is clear that EC heterogeneity supports normal physiology in organ systems with specific requirements, such as the blood-brain-barrier whose main function is protection versus the renal vasculature whose main function is filtration. Although numerous reports have indicated variable intra-organ and inter-vascular EC expression profiles, the molecular mechanisms underlying this phenomenon are not fully understood [60, 86, 187–189]. One study reported epigenetic changes across nine different EC types (3 of which were cardiac), confirming differential expression (of e.g. homeobox genes) in parallel with identification of EC-specific enhancers and markers of EC type, together with levels of active histone marks [189]. However, it is increasingly evident that DNA methylation may also be important in dictating EC regulation in specific EC populations under basal and pathological conditions, whilst noting that most of the available data for nucleic acid methylation have been produced using non-cardiac ECs, such as HUVECs or HRECs. Due to known heterogeneity of ECs, these cell lines may not be representative models for investigating DNA methylation in the context of the heart [190], so given the emerging role of ECs in HF, it is critical that focused analyses of DNA methylation are conducted across subsets of cardiac ECs. It is particularly important to assess methylation changes in ECs exposed to relevant pathophysiological stresses, such as hyperglycaemia, hypoxia, and ischaemia, and translating this to the development and clinical application of epigenetic targeting of dysfunctional ECs for HF therapy.

The attraction of these epigenetic changes from a therapeutic perspective lies in their inherently reversible nature along with potential for pharmacologically targeting and modulation. There are currently two inhibitors of DNA methylation, 5aza and 5azadC, clinically approved for treatment of haematological malignancies such as acute myeloid leukaemia and myelodysplastic syndromes. Whilst both of these agents beneficially impact pathological cardiac remodelling in preclinical models of HF [9, 50, 51], their precise mechanisms of action in this setting remains to be determined. However, the ability of these agents to influence DNA methylation in ECs both in vitro and in vivo in different vascular pathologies, suggests that they could act directly on ECs to attenuate pathological cardiac remodelling. Similarly, due to accumulating evidence supporting dysfunctional RNA methylation as a key aspect of disease pathophysiology, much effort has been directed towards the design of pharmacological agents that can actively target writer and eraser proteins mediating epi-transcriptomic modifications. Indeed, in silico discovery and subsequent characterisation has facilitated identification of small-molecule compounds that efficiently bind to and activate the METTL3-METTL14 and WTAP writer complex, leading to increased levels of m6A in cellular mRNA and rRNA [191]. In addition, drugs with known therapeutic action may also influence methylation of RNA species. For example, meclofenamate sodium, a non-steroidal anti-inflammatory agent used to inhibit prostaglandin synthesis for treatment of arthritis, also selectively inhibits FTO demethylation to increase m6A levels [192]. Similarly, in addition to inhibiting DNA methylation, 5aza can incorporate into RNA and inhibit DNMT2-mediated m5C RNA methylation [193]. While understanding of the role of RNA methylation (including both adenine and cytosine) in cardiovascular disease is increasing, further research is clearly required to define specific involvement of these epi-transcriptomic processes in different HF aetiologies to assess their precise contribution to EC dysfunction in this setting.

Although there is major therapeutic promise regarding use of apabetalone for treatment of post-acute coronary syndrome in type 2 diabetic patients with cardiovascular disease, currently under evaluation in the BETonMACE phase III trial [194], there are no specific epigenetic-modifying drugs approved for the treatment of cardiovascular diseases. Nonetheless, the exciting prospect for clinical application of epigenetic therapies for HF is increasingly propelling advancements in epigenetic drug design and delivery. These include development of novel oral formulations of 5aza [195], along with the application of enzyme inhibitors to prevent breakdown of nucleosidal analogues, which may be more convenient to use with increased drug bioavailability, which show improved outcomes in oncology patients [196]. One of the major limitations of currently approved DNMT inhibitors is their ability to reduce global DNA methylation rather than promoting demethylation at specifically targeted genomic loci. However, this is being addressed through advancements in CRISPR-Cas9 technology facilitating Cas9 fusion with epigenome-editing enzymes (e.g. TETs, DNMTs) to induce site-specific changes in methylation. This concept has demonstrated positive preliminary results in the context of diabetic kidney disease in which Cas9 fusion with *TET1* specifically reduced cytosine methylation at DMRs in the *TNF-α* gene leading to enhancement of expression [197]. As such, this approach shows great potential and could also be exploited to induce DNA methylation at specific genomic loci by coupling Cas9 to DNMT enzymes to induce methylation-specific gene silencing. There has also been major development of novel strategies for cardiac-specific drug delivery, including peptide-loaded nanoparticle delivery via inhalation. For example, cell-penetrating mimetic peptides (R7W-MP) loaded to calcium phosphate nanoparticles were shown to be more efficient for cardiac-targeted delivery than other routes of administration including oral, intravenous or intraperitoneal, resulting in improved myocardial function in a rat model of diabetic cardiomyopathy [198]. Such technology represents an exciting platform for delivery of epigenetic-modifying agents (e.g. small-molecule inhibitors, non-coding RNAs) directly to the myocardium thereby maximising their therapeutic action whilst minimising adverse off-target effects.

In conclusion, whilst the future of epigenetic therapy for HF is promising, further research is clearly required to elucidate precise underlying mechanisms, including specific involvement of nucleic acid methylation in EC dysfunction and its contribution to pathological cardiac remodelling. Answering such fundamental questions is likely to inform development of EC-targeted strategies and translation of epigenetic-modifying therapies for clinical benefit in HF.
